# Guillain–Barre Syndrome in Patients With Cystic Fibrosis: A Case Series

**DOI:** 10.1002/rcr2.70308

**Published:** 2025-08-04

**Authors:** James Nolan, Maxter Thai, Vanessa Moore, Daniel Henderson, David Reid

**Affiliations:** ^1^ Department of Thoracic Medicine The Prince Charles Hospital Brisbane Australia; ^2^ School of Medicine University of Queensland Brisbane Australia; ^3^ Queensland Institute of Medical Research Brisbane Australia

**Keywords:** cystic fibrosis, Guillain–Barre syndrome, respiratory infection

## Abstract

People with cystic fibrosis (CF) typically experience chronic respiratory infections, but neurological sequelae are rare. Guillain–Barre Syndrome (GBS) is classically precipitated by a respiratory or gastrointestinal infection, although other rarer aetiologies exist. This case series outlines four adults with CF who developed GBS. The association with acute and chronic respiratory infections in people with CF is explored, as well as other potential precipitants. An autoimmune phenomenon in the context of chronic systemic inflammation or a possible contributory role of dysfunctional CFTR protein is also considered.

## Graphical abstract

We present four cases of Guillain–Barre Syndrome in adult patients with cystic fibrosis. We highlight the particularly detrimental impact that can occur in a population of patients with chronic underlying suppurative airway disease, impaired lung function and metabolic dysregulation.

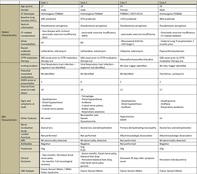

## 1 Introduction

Cystic fibrosis (CF) is an autosomal recessive disease caused by mutations in the CF transmembrane conductance regulator (*CFTR*) gene with multi‐system effects including pulmonary, gastrointestinal, endocrinological, musculoskeletal and genitourinary. The lungs are the most severely affected organ, with chronic airway bacterial infection causing progressive deterioration in lung function [1]. Rare descriptions of neurological abnormalities in CF patients, particularly peripheral neuropathy, are hypothesised to be secondary to CF‐related diabetes, micronutrient deficiencies, or due to a yet unknown immunological aetiology [2].

Guillain–Barre syndrome (GBS) is an eponym encompassing acute inflammatory demyelinating polyneuropathies (AIDP). Classically precipitated by a respiratory or gastrointestinal infection, GBS is characterised by progressive, symmetrical, flaccid muscle weakness, areflexia and ascending sensory changes. GBS occurs secondary to molecular mimicry, and the resultant cross‐reactivity of an immune response to a preceding infection with components of peripheral nerves. It is a heterogeneous syndrome with multiple variants, and the dysregulated immune response can cause both axonal loss and demyelination [3].

Here, we present four cases of GBS in adult patients with CF. We highlight the particularly detrimental impact that can occur in a population of patients with chronic underlying suppurative airway disease, impaired lung function and metabolic dysregulation.

## 2 Case Series

Table 1 provides a summary of baseline patient characteristics and clinical presentation, clinical course and treatment of GBS in the case series cohort.

**TABLE 1:** Case series summary.

		Case 1	Case 2	Case 3	Case 4
Patient Characteristics	Age (years)	27	18	54	29
	Gender	Male	Male	Female	Male
	CF genotype	Homozygous F508del	Homozygous F508del	F508del/2657+5G>A	Homozygous F508del
	Baseline lung function (FEV_1_)	40% predicted	97% predicted	< 15% predicted	98% predicted
	Sputum colonisation	*Pseudomonas aeruginosa*	*P. aeruginosa*	*P. aeruginosa*	*P. aeruginosa*
	CF‐related complications	Liver disease with cirrhosis Pancreatic exocrine insufficiency DIOS	Pancreatic exocrine insufficiency ABPA	Pancreatic exocrine insufficiency	Pancreatic exocrine insufficiency CF‐related diabetes
	Other Comorbidities	Nil	Nil	Rheumatoid arthritis CKD Stage II	Bilateral lung transplantation 2 months prior
	Recent antibiotic exposure	Ceftazidime, tobramycin	Ceftazidime, tobramycin	Cefepime, tobramycin	Piperacillin/tazobactam
	CFTR modulator therapy	GBS onset prior to CFTR modulator therapy era	GBS onset prior to CFTR modulator therapy era	Elexacaftor/tezacaftor/ivacaftor	GBS onset prior to CFTR modulator therapy era
GBS characteristics	Inciting incident	Viral Respiratory tract infection—organism not identified	Viral Respiratory tract infection—organism not identified	Nil clear trigger identified	Nil clear trigger identified
	Potential associated medications	Nil identified	Nil identified	Nil identified	Tacrolimus, cyclosporin
	EGRIS score at presentation	2	4	2	2
	Interval from onset to nadir (days)	10	10	12	17
	Signs and symptoms at nadir	Quadriparesis Distal hypoesthesia Areflexia Cranial nerve palsies	Tetraplegia Distal hypoesthesia Areflexia Cranial nerve palsies Bulbar palsy Respiratory weakness	Quadriparesis Distal hypoesthesia Areflexia	Quadriparesis Areflexia Respiratory weakness
	Other features	Nil noted	Neuropathic pain Sweats Dysautonomia	Hypertension SIADH	Nil
	Nerve conduction study	Axonal loss	Axonal loss and demyelination	Primary demyelinating neuropathy	Axonal loss and demyelination
	Lumber puncture	Not performed	Not performed	Albuminocytologic dissociation	Albuminocytologic dissociation
	MRI	Nil abnormality detected	Nil abnormality detected	Not performed	Nil abnormality detected
	Antibodies	Negative	Negative	Not performed	Negative
	Treatment	IVIg	IVIg PLEX	IVIg	IVIg
	Clinical Outcome	Two months: persistent facial nerve palsy Six months: full neurological recovery	Seven months: Facial nerve palsy, bilateral foot drop Persistent bilateral foot drop, mild facial nerve palsy persists	Deceased 39 days after symptom onset	Persistent mild dysarthria
	GBS Subtype	Classic sensori‐motor/Miller Fisher syndrome	Classic sensori‐motor	Classic sensori‐motor	Classic sensori‐motor

Abbreviations: CF, cystic fibrosis; EGRIS, Erasmus GBS respiratory insufficiency score (clinical tool to predict risk of respiratory failure in the first week after hospital admission for patients with GBS); FEV_1_, forced expiratory volume in 1 s; GBS, Guillain–Barre syndrome; IVIg, intravenous immunoglobulin; PLEX, plasma exchange.

### 2.1 Case 1

A 27‐year‐old male diagnosed with CF (homozygous for F508del CFTR mutation) was admitted to hospital with 5 days of progressive peripheral weakness, 3 days of peripheral paraesthesia and a 1‐day history of intermittent diplopia, with increasing difficulty performing airway clearance, occurring two weeks following an unidentified upper respiratory tract infection (URTI). His medical history was relevant for moderately severe CF‐related bronchiectasis, CF‐related liver disease with Child–Pugh A Cirrhosis and pancreatic exocrine insufficiency. Areflexia with progressive peripheral weakness and the development of facial nerve and bulbar weakness and bilateral diplopia were noted on presentation; however, spirometry remained stable throughout admission. Treatment with intravenous immunoglobulin (IVIg) was provided. Hospitalisation was complicated by a pulmonary exacerbation of CF, for which targeted antimicrobial therapy was provided. Standard airway clearance techniques proved difficult due to facial and bulbar weakness. Symptoms stabilised at Day 5 of admission before gradually improving until hospital discharge on Day 17. At 2 months follow‐up, mild facial nerve palsy persisted, but with complete neurological recovery evident at 6 months.

### 2.2 Case 2

An 18‐year‐old male with CF (homozygous for F508del CFTR mutation) was admitted to hospital for management of a pulmonary exacerbation of CF‐related bronchiectasis on the background of an unidentified URTI 2 weeks prior. A concomitant 3‐day history of progressive peripheral upper and lower limb weakness and paraesthesia was volunteered. Medical history was relevant for mild CF‐related bronchiectasis, pancreatic exocrine insufficiency and allergic bronchopulmonary aspergillosis (ABPA). Areflexia and rapidly progressive tetraplegia were evident on presentation, with associated bulbar weakness and declining respiratory function. IVIg was commenced, but rapidly progressive bulbar and respiratory involvement resulted in an intensive care unit (ICU) admission (on Day 3 of admission) for intubation and ventilation. During a 73‐day ICU admission, plasma exchange (PLEX) and IVIg therapy were provided and a tracheostomy was performed. Hospitalisation was complicated by severe neuropathic pain, autonomic dysfunction and pneumonia. A further 15‐day acute and 84‐day inpatient rehabilitation hospitalisation followed. On discharge, there was residual peripheral lower limb paraplegia and cranial nerve palsy, with modest improvement over 5‐year follow‐up.

### 2.3 Case 3

A 54‐year‐old female with CF (F508del/2657+5G>A) was admitted to hospital with a pulmonary exacerbation of CF‐related bronchiectasis and a 3‐day history of increasing distal paraesthesia. Her medical history was relevant for severe CF‐related bronchiectasis complicated by chronic hypoxia and hypercarbia managed with long‐term oxygen therapy (LTOT) and nocturnal non‐invasive ventilation (NIV), pancreatic exocrine insufficiency, quiescent rheumatoid arthritis (RA) and Chronic Kidney Disease Stage II. Admission neurological examination was consistent with previously documented mild lower limb peripheral neuropathy secondary to RA. Treatment for a pulmonary exacerbation with intravenous antibiotics was initiated. On Day 4 of admission, progressive lower limb, then upper limb weakness with hyporeflexia developed. Treatment with IVIg was provided. Admission was complicated by acute on chronic hypercapnic respiratory failure and *Clostridium difficile* infection. Despite improvement in peripheral neurology, the patient's respiratory status deteriorated, secondary to the pulmonary exacerbation of CF‐related bronchiectasis and she died on Day 36 of hospitalisation.

### 2.4 Case 4

A 29‐year‐old male with CF (homozygous for F508del CFTR mutation) was admitted to hospital with a one‐week history of progressive lower limb weakness and increasing falls without preceding illness. His medical history was relevant for a bilateral lung transplantation 2 months prior for severe CF‐related bronchiectasis. Neurological examination demonstrated progressive lower and upper limb weakness, with bulbar involvement. Respiratory involvement was evident on declining spirometry. IVIg therapy was provided, tacrolimus and cyclosporin immunosuppression were switched to everolimus and mycophenolate due to the potential association with GBS. A 73‐day hospitalisation was complicated by pneumonia. Neurological function improved, but at long‐term follow‐up, residual dysarthria remains.

## 3 Discussion

To our knowledge, these four cases represent the first descriptions of GBS occurring in adults with CF. Three patients developed serious short‐term sequelae, whilst one patient died as a consequence of underlying very severe CF‐related bronchiectasis compounded by exacerbation of ventilatory failure by GBS. Of the three surviving patients, all demonstrated slow neurological recovery, with two experiencing permanent neurological effects. The care for all patients was complex and required significant multidisciplinary input. Particularly challenging were the impact of a progressive neuropathy on mobility, airway clearance, nutrition and hydration; the cornerstones of CF care.

The reported incidence of GBS in Australia is 1.7 per 100,000 population/year, and our CF centre cares for approximately 350 patients across a wide geographic area. Given the dispersion in time, patient geographic residence and season at time of hospitalisation of each of the described cases, no unifying aetiology for our cohort has been identified. At our institution, most adults with CF have been diagnosed in childhood, via standard diagnostic means and initially managed through specialist paediatric CF services prior to transition to adult care [1].

Whilst there are multiple established or proposed causal associations, the majority of GBS cases occur post respiratory or gastrointestinal infections, with URTIs the probable precipitant in two of our reported cases [4]. It is notable that in two cases a clear preceding infection was not identified. Several non‐infectious aetiologies for GBS have been suggested, and a wide range of potential aetiologies were considered in each of our reported cases. Only in case Four were potential medications previously associated with GBS, tacrolimus and cyclosporin, present and thus identified as a potential cause and promptly ceased [5, 6]. The antimicrobial agent colistin has also been identified as a potential GBS mimic, but none of our patients demonstrated a temporal exposure to this or similar antimicrobial agents [7]. Finally, all patients in this case series had exocrine pancreatic insufficiency, a risk factor for vitamin and mineral deficiency, which are recognised causes of peripheral neuropathy in patients with CF [2]. It is standard practice in our centre that all patients receive regular guideline‐directed nutritional supplementation and regular monitoring to ensure against micronutrient deficiency [8]. It is also notable that there was no reported family history of GBS or other neurological disorder.

People with CF classically experience acute and chronic respiratory infection caused by a wide range of pathogens [9]. It is worth considering that CF‐associated chronic systemic inflammation, alterations in immune function and microbiological dysbiosis represent risk factors for the development of molecular mimicry and autoimmune phenomena [10]. Interestingly, the CFTR protein has a role in airway epithelial cell wound healing following injury. Due to absent or dysfunctional CFTR, there is reduced production of GM1 gangliosides, which are involved in cell–cell communication and exert anti‐inflammatory effects [11]. Anti‐GM1 ganglioside antibodies are detected in approximately 60% of patients with GBS [4]. Whether there is a link between a pre‐existing cellular deficiency of GM1 gangliosides in CF and susceptibility to neuronal injury could be theorised. However, it is notable that anti‐GM1 antibodies were not detected in any patients in our case series.

Although an association between GBS and CF has not been previously reported, this case series should focus the attention of CF physicians towards early consideration, investigation and neurological referral. A high index of suspicion could lead to earlier diagnosis and management, and subsequently improve patient outcomes. Given the observed morbidity and mortality highlighted by this case series, prompt treatment should be considered in any person with CF exhibiting a clinical syndrome concerning GBS. This case series also adds to the growing literature on the extra‐pulmonary role of the CFTR protein and CF‐related autoimmune phenomenon.

## Author Contributions

All authors contributed equally to the development of this manuscript.

## Ethics Statement

All patients provided consent, and ethics approval to report this case series was obtained from the local ethics committee (HREC/2023/MNHA/100792).

## Consent

The authors declare that written informed consent was obtained from all the patients for the publication of this manuscript and accompanying images and attest that the form used to obtain consent from the patients complies with the Journal requirements as outlined in the author guidelines.

## Conflicts of Interest

The authors declare no conflicts of interest.

## Data Availability

Data sharing is not applicable to this article as no new data were created or analyzed in this study.
